# 

**Published:** 1873-10-01

**Authors:** 


NORTH-EASTERN INDIANA MEDICAL SOCIETY.—The next meeting of the North-Eastern Indiana Medical Society will be held at Waterloo, on Tuesday, Sept. 30th, 1873.

Subject for Discussion: "The Mechanical Treatment of Uterine Displacements."

Essayists: N. Teal, J. B. Casebeer, E. G. White.

J. L. GILBERT, *Sec'y*,

